# In the last 10 years, have our polytrauma patients become geriatric? The emergency trauma bay in the context of demographic change

**DOI:** 10.1007/s00068-024-02703-8

**Published:** 2025-01-24

**Authors:** Sabrina Bindrich, Thomas Mittlmeier, Steffi S. I. Falk

**Affiliations:** Clinic of Trauma, Hand and Reconstructive Surgery, University of Rostock, Schillingallee 35, 18055 Rostock, Germany

**Keywords:** Traumatology, Polytrauma, Geriatric patients, TR-DGU, Trauma center

## Abstract

**Purpose:**

One of the key challenges trauma centres are currently facing is the management of polytraumata in an ageing population. The aim of this study is to assess the extent to which demographic changes are reflected in the trauma bay population and the impact on geriatric polytrauma patient outcomes.

**Methods:**

This is a retrospective single-centre cohort study of a level one trauma centre in Germany. The data were collected from the DGU TraumaRegister. All patients exhibiting vital signs who were primarily admitted to the trauma bay were included in the study. Patient characteristics were compared for years 2011 and 2021. Polytrauma was defined as ISS > 15, and patients aged 65 and over were assigned to the geriatric group.

**Results:**

The study included 214 patients. During the study period, there was a significant increase in the mean age of patients (from 47.7 to 55.9 years) and in the proportion of geriatric patients (from 30.37 to 40.51%). Injury severity, as measured by the Injury Severity Score (ISS), also increased significantly. In the entire patient population, the proportion of patients discharged to their place of residence decreased, while the hospital mortality (2011: 9.63%; 2021: 21.52%) increased.

**Conclusions:**

The ageing trauma bay population presents new challenges for medical staff, because polypharmacy, multiple comorbidities and frailty become more significant in an ageing population. Enhanced interdisciplinary management, particularly between trauma and geriatric specialists, may mitigate rising mortality rates. Geriatric trauma centres or at least more geriatric expertise might be required to improve the treatment and outcome in this changing population.

## Introduction

The care of patients who have sustained severe injuries represents one of the primary responsibilities of trauma surgery [1]. Already in 1993, the German Trauma Society (DGU) established the nationwide TraumaRegister, which serves as a quality management tool and has since become one of the largest registries of its kind in the world [2, 3]. Today, in the context of demographic change, it is necessary to consider the potential impact on the patient collective in the trauma bay [4‑8].

The present study employs a retrospective cohort design to examine patients admitted via the trauma bay of the Rostock University Medical Centre in the years 2011 and 2021. The data for this study were obtained from the TraumaRegister DGU. The Rostock University Medical Centre is one of three level one trauma centres in the federal state of Mecklenburg-Vorpommern (MV). As of 2022 Mecklenburg-Vorpommern is the federal state with the lowest population density in Germany [9]. Concurrently, the proportion of the population aged 65 and above is above the national average, as evidenced by data from the Federal Statistical Office [10]. As reported by the Federal Statistical Office, it is also the federal state with the most rapid ageing population, thus representing an optimal model for analysis [11]. These circumstances present acute challenges in the care of severely injured geriatric patients for hospitals in MV, and may offer insights for other regions.

Several retrospective analyses of the TraumaRegister have demonstrated an increase in the age of patients since the register was established. At the same time, there has been a reduction in hospital mortality and a decrease in the length of stay in hospital and in the intensive care unit. Additionally, there was a shift in the type of accident, with an increase in falls from a height of less than three metres, which represents the primary cause of injuries in older patients [4, 5]. Furthermore, the increasing age of severely injured patients has been observed internationally in several studies [6‑8]. Consistent with the previously mentioned studies the authors hypothesise that the demographic change will also have an impact on patients in the trauma bay, resulting in a notable increase in the number of geriatric trauma patients admitted to the hospital via the trauma bay in 2021 compared to 2011. In addition, Braun et al. [12] suggest that comorbidity is an independent risk factor for mortality in the elderly and the chances of injury as a result of falls and car accidents increase with age. Therefore, it is anticipated that mortality and the length of hospitalisation will increase in this age group due to the greater number of comorbidities, whereas mortality across all age groups is expected to decline. In line with the studies mentioned above it can furthermore be hypothesised that the accident mechanisms and resulting injury patterns are shifting from road traffic accidents to lower energy traumas, such as falls from low heights.

## Methods

The registry study presented here is a retrospective, single-center cohort study of a level one trauma center. The data set was primarily assembled from the TraumaRegister of the DGU, with any missing data subsequently supplemented from the patient files. The periods from 1 January 2011 to 31 December 2011 and from 1 January 2021 to 31 December 2021 were compared. The participation in the TraumaRegister resulted in the mandatory entry of all patients meeting the inclusion criteria for the TraumaRegister starting in 2010. Therefore, the authors decided to choose the year following this implementation as the earliest possible year. By doing so, the in-hospital organizational structures and compliance with TraumaRegister requirements could be considered well-established. With the study being planned in 2022, the question arose whether to include a period of 11 or 10 years. The authors opted for 10 years to ensure better comparability. This study focused on a number of key parameters, including gender, age, Injury Severity Score (ISS), New Injury Severity Score (NISS), American Society of Anesthesiologists risk classification (ASA score), admission time, type of accident, injury pattern, duration of intensive care, and duration of mechanical ventilation. Additionally, the outcome of each case was recorded, including whether the patient was discharged from the hospital or transferred to another facility. The patients were discharged to: home, a rehabilitation clinic, another hospital, or another rehabilitation clinic. The discharge status was recorded as: well recovered, moderately disabled (disabled but independent), severely disabled (conscious but disabled and dependent on assistance [13]), unresponsive, or deceased. In this study mortality is defined as hospital mortality and was recorded upon discharge of the patients. The length of stay in the hospital was also documented.

The demographic data for Germany and Mecklenburg-Vorpommern is obtained from the website of the Federal Statistical Office [10]. The reference dates used are the 31st of December, 2011 and the 31st of December, 2021.

### Patients

The TraumaRegister DGU includes all patients admitted to one of the participating hospitals via the trauma bay with documented vital signs who subsequently require intensive care, undergo transfer to the operating theatre, or die before admission to the intensive care unit. Furthermore, in accordance with the EU General Data Protection Regulation (GDPR) that acquired legal force in May 2018, a declaration of consent in the form of a standardised information sheet must be provided for each patient [13, 14]. Patients transferred from another hospital to the trauma bay and thus not primarily admitted to Rostock University Medical Center are excluded from this study, as the authors lack complete data for this patient group. This study focuses particularly on the subgroup of geriatric polytrauma patients. Polytrauma was defined as an ISS > 15, and patients aged 65 and over were categorized as geriatric.

### Statistical analysis

The statistical analysis was conducted using IBM SPSS Statistics version 29. To compare the two study periods, both the Mann-Whitney U test for independent samples and Pearson’s chi-square test were employed. The level of statistical significance was set at 5% (α-error = 0.05). The continuous and categorical variables such as mean, count, standard deviation and percentage, were calculated with using Microsoft Excel 2019 Version 1808.

## Results

**Fig. 1:**
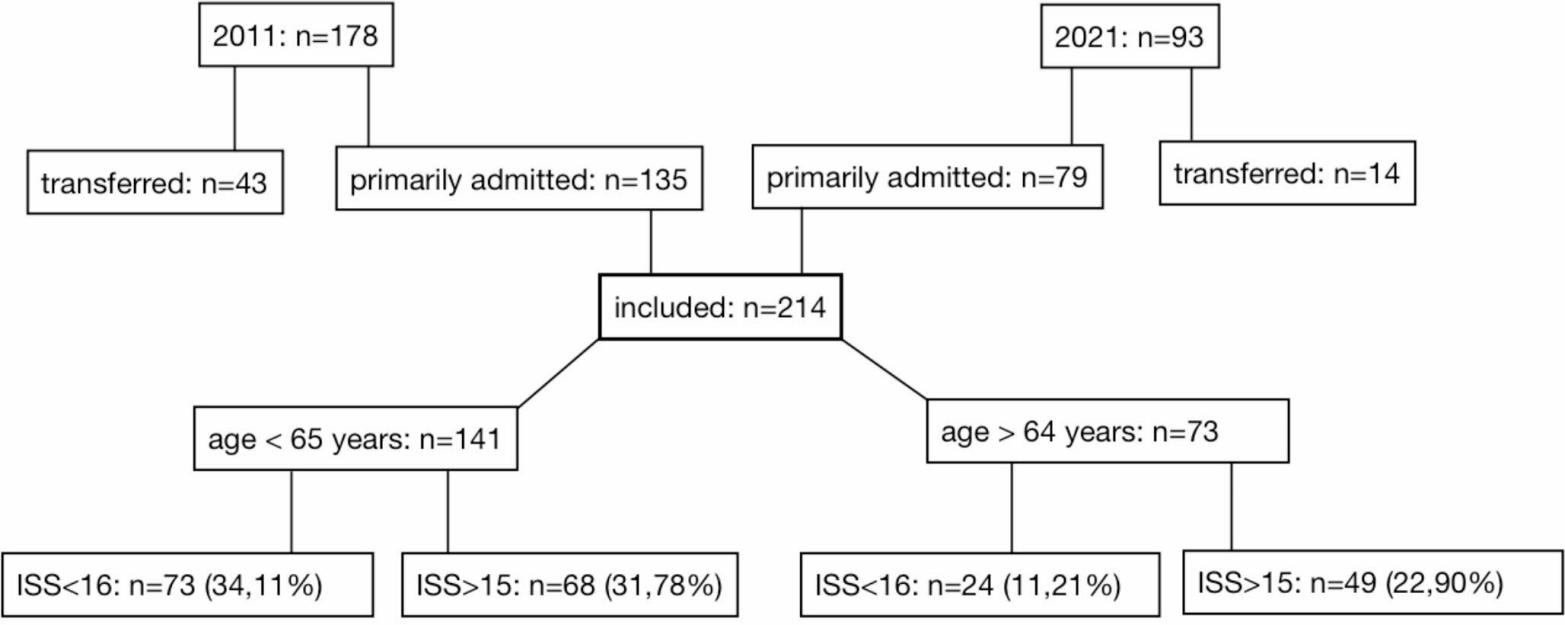
provides an overview of the study population, including the division into the subgroups investigated

Between 1 January 2011 and 31 December 2011 (the 2011 period), 178 patients were recorded in the TraumaRegister DGU database. Of these, 43 were transferred patients and therefore excluded from the analysis. In the period between 1 January 2021 and 31 December 2021 (the 2021 period), a total of 93 patients were recorded, of whom 14 were transferred (see Fig. 1). Thus the patient population under consideration in this study consisted of 214 patients (135 in the 2011 period and 79 in the 2021 period). The data for age, gender, admission time, ISS, NISS, length of hospital stay, duration of intensive care and injury pattern were fully complete. The data was 98.1% complete for the American Society of Anesthesiologists (ASA) score, 96.3% complete for the duration of ventilation, 97.2% complete for the outcome, 99.5% complete for the condition at discharge, and 99.1% complete for the accident type.

The number of patients decreased during the study period, from 135 in 2011 to 79 in 2021. Concurrently, the proportion of geriatric patients (aged 65 years or older) increased, from 30.37% in 2011 to 40.51% in 2021. A notable increase in age was observed over the course of the study (p-value: 0.018). The mean age increased from 47.73 years in 2011 to 55.92 years in 2021, indicating that the average age of patients in both study periods was significantly higher than the national average and the average age in Mecklenburg-Vorpommern. Furthermore, the proportion of geriatric patients was higher than that observed in the overall German and Mecklenburg-Vorpommern populations (see Table 1). This increase was disproportionate in relation to the total population during the study period.

**Table 1:** Comparison of the demographic data for the two study periods, with the respective demographic data for Germany and Mecklenburg-Vorpommern (MV)

	2011	2021
Average age of patients (years)	47.7 (22.34–73.12)	55.9 (31.91–79.93)
Average age in MV (years)	45.8	47.5
Average age in Germany (years)	43.9	44.7
Proportion of patients aged ≥ 65 (%)	30.37	40.51
In MV (%)	22.09	26.26
In Germany (%)	20.70	22.15
Propotion of male patients (%)	67.41	81.01
In MV (%)	49.26	49.25
In Germany (%)	48.84	49.34

Additionally, there was an observed increase in the proportion of male patients. In 2011, 67.41% of patients were male, whereas in 2021 this percentage had risen to 81.01%. This indicates that the proportion of male patients was greater than the proportion of males in the overall population of Germany and Mecklenburg-Vorpommern (see Table 1). Moreover, this increase was disproportionate when compared to the total population. Furthermore, the severity of injury, as indicated by the Injury Severity Score (ISS) and the New Injury Severity Score (NISS), increased significantly over the observation period (see Table 2). The duration of mechanical ventilation increased significantly (p-value: 0.004; 2011: 76.80 h; 2021: 111.41 h) as well. No significant differences were observed between the two study groups with regard to pre-existing conditions (ASA score; p-value: 0.973), duration of intensive care (p-value: 0.115) and length of hospital stay (p-value: 0.125).

A significant increase in ISS and NISS was observed in the geriatric patient population, as well as in the overall patient population (see Table 2). No significant differences were observed between the two study periods with regard to ASA score (p-value: 0.119), admission time (p-value: 0.377), age (p-value: 0.585), duration of intensive care therapy (p-value: 0.862), length of hospitalisation (p-value: 0.410) or duration of mechanical ventilation (p-value: 0.646) within this subgroup. In the subgroup of geriatric polytrauma patients, a significant reduction in the duration of intensive care (p-value: 0.007), the duration of mechanical ventilation (p-value: 0.011) and the total length of hospitalisation (p-value: 0.030) was observed (see Table 3).

**Table 2:** Comparison of injury severity measured by ISS & NISS: mean value (standard deviation)

	2011	2021	*p*-value
ISS total collective	15.56 (3.41–27.71)	22.78 (12.46–33.10)	0.001
ISS geriatric subgroup	16.61 (4.04–29.18)	22.72 (15.11–30.33)	0.004
NISS total collective	19.36 (4.19–34.53)	29.96 (15.72–44.20)	0.001
NISS geriatric subgroup	22.02 (4.58–39.46)	31.56 (19.99–43.13)	0.001

**Table 3:** Characteristics of patients in the intensive care unit for the subgroup of geriatric polytrauma patients (ISS > 15, age ≥ 65 years), both for 2011 and 2021. The data are presented as mean (standard deviation), with duration in days, and mechanical ventilation duration in hours

	2011	2021	*p*-value
Duration Intensive Care Unit therapy	13.68 (3.05–24.31)	5.50 (-1.74-12.74)	0.007
Duration of mechanical ventilation	234.95 (-11.31-481.21)	65.47 (-63.19-194.13)	0.011
Length of hospital stay	18.53 (5.31–31.75)	11.97 (0.81–23.13)	0.030

A comparison of the entire patient population revealed a statistically significant decrease (p-value: 0.114) in the proportion of patients discharged to home in 2021 (53.16%) compared to 2011 (62.22%), while hospital mortality increased (2011: 9.63%; 2021: 21.52%). The proportion of patients transferred to another hospital (2011: 5.19%; 2021: 3.80%) or a rehabilitation clinic (2011: 20.00%; 2021: 18.99%) remained relatively constant.

A significant increase in hospital mortality was observed in geriatric patients as well as in the overall collective (2011: 14.63%; 2021: 40.63%). There has been a decline in the number of discharges to the patients’ place of residence (2011: 53.66%; 2021: 43.75%) and transfers to a rehabilitation clinic (2011: 26.83%; 2021: 6.25%), as evidenced by a p-value of 0.097. In contrast, a notable increase was observed in discharges to home for geriatric polytrauma patients (2011: 26.32%; 2021: 46.67%), accompanied by a pronounced decline in transfers to rehabilitation facilities (2011: 47.37%; 2021: 3.33%) (p-value: 0.871). The in-hospital mortality rate for geriatric polytrauma patients increased significantly, from 26.32% in 2011 to 43.33% in 2021. The overall hospital mortality rate increased significantly, from 9.63% in 2011 to 21.52% in 2021. The greatest increase was observed in the geriatric age group, which also exhibited the highest mortality rate in both periods (see Table 4).

**Table 4:** Hospital mortality in the individual subgroups

	2011	2021	*p*-value
Overall	9.63%	21.52%	0.114
Age 0–18	4.17%	20.00%	0.061
Age 19–64	8.45%	7.32%	0.676
Age ≥ 65	14.63%	40.63%	0.097
ISS > 15	22.22%	26.98%	0.283
ISS > 15 age 0–18	25.00%	20.00%	0.796
ISS > 15 age 19–64	19.35%	10.71%	0.076
ISS > 15 age ≥ 65	26.32%	43.33%	0.871

With regard to the condition of patients upon discharge, there was a notable increase in the number of cases where patients were discharged from hospital in a poor condition, defined as cases where patients were severely disabled or unresponsive. Conversely, the proportion of patients who were well recovered or moderately disabled decreased during the study period (see Table 5).

**Table 5:** Condition of patients at discharge. The p-value for this variable is 0.032. The difference between patients who are moderately and severely disabled is defined by whether the patient is dependent on assistance in daily living

	2011	2021
Patient deceased	9.63%	21.52%
Well recovered	42.96%	35.44%
Moderately disabled	39.26%	27.85%
Severely disabled	7.41%	10.13%
Unresponsive	0.74%	3.80%

With regard to the type of accident, the overall distribution remained approximately constant between the two study periods. The distribution of accident types among geriatric patients also remained almost unchanged. There was a slight tendency towards traffic accidents and falls from a height of at least three metres, as well as a decrease in falls from low heights. In the subgroup of polytraumatised geriatric patients, there was an increase in traffic accidents (2011: 15.79%; 2021: 30%), while there was a tendency for falls to decrease.

The highest proportion of road traffic accidents occurred among the 19–64 age group in 2011. This was also observed in the subgroup of polytrauma patients. In contrast, the highest proportion of road traffic accidents was observed among paediatric patients in 2021 (see Table 6). Moreover, the number of road traffic accidents involving members of the geriatric population rose significantly. Conversely, the proportion of falls from low heights was highest in the geriatric group, a finding that was also observed in the polytrauma patients. Nevertheless, the incidence of falls from low heights declined among the geriatric patient cohort, both in the general population and in the polytraumatised population.

**Table 6:** Type of accident in the subgroups

	Road traffic accidents		Fall < 3 m height		Fall ≥ 3 m height	
	2011	2021	2011	2021	2011	2021
Overall	44.44%	40.51%	34.81%	36.71%	12.59%	17.72%
Age 0–18	37.50%	80.00%	29.17%	20.00%	12.50%	0.00%
Age 19–64	56.34%	43.90%	19.72%	24.39%	14.08%	24.39%
Age ≥ 65	26.83%	28.13%	63.41%	59.38%	7.32%	9.38%
ISS > 15	40.74%	36.51%	42.59%	42.86%	9.26%	19.05%
ISS > 15 age 0–18	25.00%	80.00%	50.00%	20.00%	0.00%	0.00%
ISS > 15 age 19–64	58.06%	35.71%	25.81%	28.57%	6.45%	32.14%
ISS > 15 age ≥ 65	15.79%	30.00%	68.42%	60.00%	15.79%	10.00%

The data revealed a significant increase in the prevalence of thoracic injuries, from 36.30% in 2011 to 55.70% in 2021. The results of the chi-square tests for the parameters outcome (p-value: 0.114), accident type (p-value: 0.531) and injury pattern were, with the exception of thoracic injuries, not statistically significant.

## Discussion

As hypothesised, there was a notable increase in the age of patients during the study period. Furthermore, there was a decreasing length of stay in the hospital and in the intensive care unit. These findings are consistent with the results of other retrospective analyses conducted in Germany by the TraumaRegister DGU^®^ [4] and by Kalbas et al. [5]. They are also indicative of the general observed trend towards shorter periods of hospitalisation. This trend can possibly be explained by the increasing cost containment in health care, with particular consideration given to the increasingly limited number of intensive care beds. Another factor is the increasing importance of patient autonomy, which is reflected in the inclusion of this aspect in the data collection form of the TraumaRegister, leading to earlier palliative care. Moreover, the disproportionately high proportion of geriatric patients in relation to the demographic data has been demonstrated in other studies [15]. Drawing on Brown et al. [12] the authors attribute this to the increasing frailty associated with advanced age, as well as the prevalence of multimorbidity and the associated polypharmacy, which can increase the risk of falls and other adverse outcomes. The prevalence of osteoporosis, which is more common in older age groups, also increases the risk of serious injuries even in the case of minor trauma. Furthermore, reflex speed, hearing and eyesight decline, thereby reducing the ability to recognise and avert dangers in road traffic or other hazardous situations Improved medical care, however, enables many elders to remain active into advanced age, which in turn increases the probability of sustaining injuries.This may explain the observed increase in the proportion of geriatric patients in the patient population as well as the increase in injury severity. Furthermore, the authors contend that the notable increase in road traffic accidents among geriatric patients reflects an increase in the independence of the elderly [12].

With respect to the overall reduction in patient volume, it should be noted that the second study period (1 January 2021-31 December 2021) coincides with the onset of the SARS-CoV-2 pandemic and the subsequent implementation of lockdown measures in Germany.

In everyday clinical practice, it has been demonstrated that the male gender predominates in polytrauma patients [4]. Given the observed increase in road accidents, it was to be expected that there would be an increase in the number of men in the collective, however, not to the extent observed here. The DEKRA Road Safety Report (2011) indicates that, up to the age of 65, male drivers are more likely than female drivers to be involved in road traffic accidents. After that age, the proportion of both genders involved in such accidents evens out [16].

In contrast with the anticipated outcome, the mortality rate within the hospital setting increased during the course of the study. However, this is largely attributable to the disproportionate rise in mortality among the geriatric population in comparison to other age groups. The observed increase in mortality can be attributed to the elevated severity of injury, as quantified by the Injury Severity Score (ISS) and the New Injury Severity Score (NISS), among geriatric patients admitted during 2021. Consequently, the number of discharges to home or rehabilitation facilities was also reduced in this age group. The increasing severity of injury across all age groups also accounts for the observed trend towards worse conditions on discharge.

Additionally, the study data showed a rise in the incidence of thoracic injuries during the study period, a phenomenon that has been previously documented in the literature. Koch et al. [17] attribute this to some extent to an increase in the rate of whole-body CT scans performed during the study period. In a retrospective study on thoracic injuries in polytrauma patients, Chrysou et al. [18] demonstrated that road traffic accidents were the primary cause of such injuries. Therefore, the observed increase in traffic accidents may also contribute to the rising incidence of thoracic injuries. In addition, Bayer et al. [19] demonstrated that mortality is higher in polytrauma patients with thoracic injuries. We suggest that elevated incidence of thoracic injuries in 2021 could also be a relevant factor for the higher mortality rate observed during the latter period.

It is notable that the increase in mortality observed in all age groups was initially anticipated to be specific to the geriatric population. It is possible that the rise in mortality is attributable to enhanced pre-hospital care and more rigorous admission criteria due to a reduction in intensive care unit capacity, resulting in fewer and more severely injured patients being admitted to the trauma bay. An alternative hypothesis is that there has been an increase in the number of treatment limitations, particularly in the geriatric population. A further analysis of this hypothesis was not feasible due to the absence of data from the 2011 period.

To address the increasing patient age and rising hospital mortality, the implementation of certified orthogeriatric trauma centres should be considered. According to Fröhlich et al. this may improve the outcome in geriatric patients while the effectiveness of geriatric consultations alone is controversial [15]. Halvachizadeh et al. conclude that, following the establishment of geriatric comanagement, more patients were discharged home, the length of hospital stay decreased, and fewer patients required rehabilitation afterward. Despite increasing medical complexity, mortality remained unchanged [20]. The latter was also observed by Laubach et al. subsequent to certification as a geriatric trauma centre [21].

### Limitations

Given the retrospective nature of the TraumaRegister DGU and the study design, it is not feasible to derive causal inferences from the data. Additionally, the registry does not document preclinical fatalities, which introduces an element of selection bias. Another limitation is the potential inaccuracy or incorrect data entry in the TraumaRegister [22]. Additionally, the sample size is relatively limited, which impacts the statistical power of the analyses. It is also important to note that the Injury Severity Score (ISS) is not a reliable indicator of the severity of injury in patients, as evidenced by several studies [23, 24]. It is therefore recommended that the Berlin definition be used, as it is more objective because of its additional use of physiological parameters, thus reducing interobserver variability [24, 25]. However, as ISS > 15 is utilised as a threshold in the majority of studies examining polytraumatised patients, this definition has also been employed in the present analysis, thus facilitating comparisons with previous studies. For the same reason the threshold for the geriatric population in this study is still 65 years [12, 15, 26], despite patients that age often being very fit physically nowadays. Moreover, it cannot be ruled out that older individuals with traumatic injuries are also under-triaged in the authors’ hospital and that the actual number of such cases would be higher. It has been demonstrated in previous studies that the utilisation of standard trauma scores in geriatric patients results in an under-triage of patients [12, 27, 28]. It is therefore recommended that specific scores and triage criteria for the geriatric population be considered.

## Conclusions

The ageing population is also reflected in the patient population of our trauma bay, with the result that we now see older patients with more serious injuries, including those from road traffic accidents. These patients have a higher mortality rate, but if they survive, they can be discharged home more often than was the case 10 years ago. This presents new challenges for medical staff, because factors such as polypharmacy, multiple comorbidities and frailty assume greater significance in this population. Furthermore, the rise in age and severity of injury is accompanied by an increase in hospital mortality. This could be addressed by enhanced interdisciplinary management between trauma surgeons and geriatricians, among other measures. The authors therefore believe that geriatric trauma centres, or at least more geriatric expertise, are needed to improve treatment and outcomes in this ageing population.

## Data Availability

The data that support the findings of this study are not openly available due to reasons of sensitivity and are available from the corresponding author upon reasonable request.
